# Child’s play: A computational method for generating child-specific valence norms

**DOI:** 10.3758/s13428-026-02978-2

**Published:** 2026-04-13

**Authors:** Katharina Gloria Hugentobler, Astrid Haase, Jana Lüdtke, Sascha Schroeder

**Affiliations:** 1 Educational Psychology, Georg-August-Universität Göttingen, Waldweg 26, Göttingen, 37073 Germany; 2 Experimental and Cognitive Neuropsychology, Freie Universität Berlin, Halbelschwerdter Allee 45, Berlin, 14195 Germany

**Keywords:** Affective norms, Valence ratings, Vector space models, Lexical decision task

## Abstract

Research on affective processing depends on the availability of word norms. Norms for adults are widely available and can be computed for whole-language vocabularies by mapping semantic similarities from a semantic vector space to a list of label words. While such methods are abundantly studied for adults, comparable methodologies for children are few and far between. In this article, we explored whether computational methods can be used to estimate word norms for children using valence as an example. We systematically investigated the impact of two main methodological choices relevant for the computation: the source of the semantic space used to measure semantic distance and the selection of the words in the label list. In Study 1, we correlated the computational estimates from all combinations with valence ratings from adults and children. Our results indicate that norms derived from adult vector space models and label lists outperform other combinations. Norms computed based on these parameters correlate with children’s ratings at $\boldsymbol{r =.71}$ and with adults’ ratings at $\boldsymbol{r =.74}$. In Study 2, we used estimates and human ratings to predict valence effects in a lexical decision task. Again, we found that norms derived from vector space models and label lists for adults outperformed other combinations and approximated the functional form of children’s and adults’ valence effects best. We discuss the practical implications of these findings. Additionally, a new set of valence ratings from children (mean age = 12.5 years) for 535 German words is made available.

## Introduction

Word processing is affected by variables such as word length (New et al., 2006) and word frequency (Brysbaert et al., 2018), but also semantic variables such as imageability, arousal, and valence (Kuperman et al., 2014).

The influence of semantic variables on processing speed is often analyzed using metrics of lexical processing, such as naming accuracy and reaction time in a lexical decision task. In these studies, word valence typically leads to faster reaction times for both positive and negative words in comparison to neutral ones (Citron et al., 2014) while arousal is mostly reported to decrease reaction times (Larsen et al., 2008; Schwanenflugel & Stowe, 1989).

Studies on the effects of semantic variables on lexical processing typically make use of human ratings. However, computational methods as a rater-independent alternative to quantify semantic word norms (Jacobs & Kinder, 2020) are becoming more important. Although there are several different approaches for the computation of word norms (Bestgen & Vincze, 2012; Charbonnier & Wartena, 2020; Keuleers & Hronský, 2022; Recchia & Jones, 2009), one of the most commonly employed approach is proposed by Turney and Littman (2003), who offer a bootstrapping algorithm based on semantic similarities between a target word and a list of prototypical words for any given semantic dimension (Köper & Schulte im Walde, 2016).

However, all current computational approaches reported in the literature are focused on generating norms for adults, and it is unclear whether they generalize to children and developing readers. As a consequence, researchers interested in semantic processing in children have to rely on child ratings collected for a small number of words on a few select dimensions.

In the present study, we will explore whether computationally derived word norms can also be used for children and how these norms should be computed. As most research on semantic processing in children and most databases of child-ratings focus on word valence, we will discuss the application of this algorithm to the generation of child-appropriate valence norms as an example.

Specifically, we will systematically investigate the impact of two main methodological choices relevant for the approach proposed by Turney and Littman (2003): (a) the source of the semantic space used to measure semantic distance and (b) the selection of prototypically positive and negative words used for evaluating the valence of a target word (label list).

In Study I, we compare all computed norms with human ratings from children and adults to evaluate which combination of Vector Space Models (VSMs) and label lists yields norms that correlate best with child ratings. In Study II, we will examine which combination of Vector Space Models (VSMs) and label lists is best suited to compute norms to approximate the relationship found between word valence (human ratings) and response times in lexical decision tasks across both children and adults.

### Semantic processing in adults and children

Psychological research focusing on affective processing of spoken/written language is hinged on the availability of word norms. Following the publication of the ANEW database for English words (Bradley & Lang, 1999), the availability of datasets with such types of human ratings for adults increased, and rating databases for many different languages were published (e.g. English: Warriner et al., 2013, German: Võ et al., 2009, French: Monnier & Syssau, 2014, Italian: Montefinese et al., 2014, Dutch: Moors et al., 2013, Spanish: Stadthagen-Gonzalez et al., 2017, Chinese: Yao et al., 2017, Finnish: Eilola & Havelka, 2010, and Polish: Imbir, 2016). This has significantly enriched our understanding of how adult readers engage in the cognitive and affective processing of verbal stimuli (Citron et al., 2014; Hinojosa et al., 2020).

In this context, the best-studied semantic variables are valence, arousal, and imageability. Research into affective text processing has found a processing advantage for positive and negative compared to neutral words with data on reaction times in single word lexical decision tasks (Citron et al., 2014; Kuperman et al., 2014), written and aural single word memory tasks (Arriagada-Mödinger & Ferreira 2022; Barriga-Paulino et al., 2022), and finally processing as well as memory effects on the text level (Arfé et al., 2023; Megalakaki et al., 2019). Effects of word arousal and word imageability are less explored (see Jacobs et al., 2015; Recio et al., 2014; Kuperman et al., 2014, for effects of word arousal and Dymarska et al., 2023; Garbarini et al., 2020; Westbury & Moroschan, 2009, for effects of word imageability).

Like its counterpart in adults, research into affective processing in children depends on the availability of tailored word norms for children. Recent studies concentrating on the affective processing of verbal stimuli in children have unveiled processing disparities in comparison to adults (Sylvester et al., 2021). These studies, however, face a limitation in terms of the number of words for which age-appropriate rating data are available and therefore often rely on norms collected or computed for adult readers (Braginsky et al., 2019; Kim et al., 2020).

Although the first child-specific word norms can be traced back to Di Vesta and Walls (1970), who collected ratings for English on child-specific semantic categories (e.g., brave - not brave, big - small), word norms for children are rather sparse and typically focus on collecting ratings for word valence. In addition, the number of words in these databases is typically very limited. For example, Vasa et al. (2006) collected valence ratings for *n* = 81 English words, Belmon et al. (2023) for *n*=180 French words, and Sylvester et al. (2016) for *n* = 102 German words. The only more extensive database has been provided by Sabater et al. (2020), who collected ratings for *n* =1400 Spanish words.

In general, the paucity of word rating data tailored to children in terms of vocabulary, frequency and relevance is a constraining factor for studies focusing on reading comprehension in early readers. Consequently, a limit is placed on the output and dissemination of such research.

### The advantage of computational methods for the generation of word norms

While the method of collecting human rating data yields norms with high validity, it is time-consuming and limited by the attention span of the raters. Except for the rating database from Warriner et al. (2013), which contains ratings for 14,000 words, most adult rating databases cover only a fraction of the full vocabulary of a language. This limitation becomes more pronounced in languages like German with their abundance of compound words^1^ and underlines the need for more efficient and comprehensive approaches to gathering linguistic data.

Computational methods to quantify the semantic properties of words can be applied to a much larger set of words. These methods typically use human data for a smaller set of words and, based on this information, extrapolate the corresponding values for a larger set of new words that have not been rated. Early approaches relied on linguistic resources (e.g. Bestgen & Vincze, 2012; Liu et al., 2014) or estimated semantic similarities based on vast association databases (Van Rensbergen et al., 2016; Vankrunkelsven et al., 2022). More recent approaches typically use the power of semantic vector space models in which a multi-dimensional vector in semantic space represents the meaning of a word. These semantic vector spaces can be generated using different methods such as count and predict models (Mandera et al., 2017; Recchia & Jones, 2009), or transformer-based neural networks (Plisiecki & Sobieszek, 2023).

Despite the array of methods available for bootstrapping norms, one of the most commonly used methods is based on the algorithm proposed by Turney and Littman (2003). It is built on the premise that words with greater cosine similarity to prototypically positive or negative words are more likely to exhibit positive or negative valence, respectively. The logic of the algorithm proposed by Turney and Littman (2003) depends on two lists of words, one containing a sufficient number of words with positive valence and the second one containing a sufficient number of words with negative valence. We will refer to these lists of prototypical positive and negative words as “label lists” throughout this publication. The quality of predictions depends on the number of words in the label list (Köper & Schulte im Walde, 2016). Typically, researchers depend on label lists comprising 40 (Köper & Schulte im Walde, 2016) to 60 (Jacobs, 2019) words. The literature offers a vast set of methods for extracting label lists ranging from a purely numerical approach (Köper & Schulte im Walde, 2016) to a purely content-driven approach as proposed by Bestgen and Vincze (2012) and Westbury et al. (2006). Furthermore, Lüdtke and Hugentobler (2023) have shown that label lists can also be directly extracted from human rating databases following a selection process based on typicality and frequency.

Thus, given a suitable representation of semantic similarities and a set of label lists, ratings for virtually any semantic category can be estimated for virtually any word (but see Snefjella & Blank, 2020 for potential pitfalls). As a consequence, a wealth of computed ratings, predominantly for German, English, and Spanish, on several psycholinguistic dimensions, such as valence, but also arousal and imageability (Bestgen & Vincze, 2012; Köper & Schulte im Walde, 2016) are now available for adults.

### Challenges involved in the computation of word norms for children

The computation of child-specific word norms follows the same rationale as the process outlined for adults above. However, contrary to the computation of adult norms, where the components required within the logic of the algorithm proposed by Turney and Littman (2003, i.e., a VSM and label lists) are easily accessible, it is unclear how the corresponding elements should be chosen for children.

A first key question is whether a child- or adult-specific vector space model should be used. Semantic spaces for children and adults differ in terms of size and specificity of the underlying corpora.

Two alternative scenarios are plausible: On the one hand, there might be an advantage of using child-specific VSMs, i.e., using a child-specific training corpus reflecting the knowledge and unique linguistic environments of children (Günther et al., 2019). However, child-specific corpora are usually rather small (comprising only a few million tokens, cf. Schroeder et al., 2015; Landauer et al., 1998) and might not allow for generating semantic spaces of sufficient quality. On the other hand, there might be an advantage of using adult-specific VSMs, which are generated using much larger language corpora (usually containing several billion tokens, cf. Baroni et al., 2014; Ferraresi et al., 2008). However, due to the content that is used to train these models (i.e., data from Wikipedia, internet crawls, etc. Grave et al., 2018; Bojanowski et al., 2017), the resulting vector spaces might be less appropriate to describe the semantic dimensions relevant for children. In light of this, the first question the present study addresses is whether a child- or an adult-specific vector space model should be used to generate semantic word norms for children.

A second key question concerns the method for selecting the label lists used in the algorithm proposed by Turney and Littman (2003). More precisely, it addresses the question of whether the selection should be based on word ratings for children or adults. Again, each option might have specific advantages and disadvantages:

**Table 1:** Design matrix: Sources for the generation of child-specific valence norms

	Vector Space	
Labels based on ...	CHILD	ADULT
child ratings of child words (child)	CHILD/child	ADULT/child
adult ratings of child words (adult_ch)	CHILD/adult_ch	ADULT/adult_ch
adult ratings of adult words (adult)	CHILD/adult	ADULT/adult

Note: All possible combinations of VSMs and rating source used to bootstrap valence norms for children according to the algorithm proposed by Turney and Littman (2003)

First, the label lists can be selected based on ratings from children for words that are child-appropriate, i.e., occur frequently in child-specific corpora. Given that raters are children and words are child-specific, these ratings might have a special advantage for the computation of child-specific word norms. However, as discussed above, databases comprising child ratings are usually very small and only cover a small fraction of the vocabulary. In addition, it might be more difficult for children to evaluate the semantic properties of a word, yielding ratings that are less reliable or have lower selectivity.

Second, the label list can be selected based on ratings from adults for words that are appropriate for adults, i.e., occur frequently in adult-specific corpora. As databases for adults are usually much larger than those for children, it is thus possible to cover a wider range of a semantic category, which might enhance the accuracy of the resulting norms. In addition, the correlations between ratings from adults and children are usually rather high (Sylvester et al., 2016). However, this expansion comes at the expense of specificity to the target audience; that is, the resulting labels might be less representative of children’s everyday language use.

The third possible strategy is to combine the advantages of the two previous strategies: The labels are selected based on ratings from adults, but the words that are rated are selected from a child-specific corpus. Thus, ratings from adults, which are easier to collect and more reliable, can be used while the rated words are still appropriate for children. The second central question addressed in the present study is thus whether there might be advantages associated with using ratings from adults or children and whether a child- or adult-specific rating database should be used to select the labels.

### The current study

In sum, there is an urgent need in the developmental literature to compute semantic norms appropriate for children. However, in contrast to adults, it is not clear how these norms should be computed. In the present study, we will systematically explore the impact of two key methodological decisions relevant to the algorithm proposed by Turney and Littman (2003): First, does the nature of the underlying vector-space model have an impact on the quality of the computed norms, that is, should a child- or adult-specific vector-space model be used? Second, how should the list of target labels used in the algorithm be selected? That is, should the selection be based on ratings from adults or children, and should they only comprise child-specific words?

The design for this study is summarized in Table 1: we will use two different vector space models (CHILD: a vector space model based on a child specific corpus, ADULT: a vector space model based on an adult specific corpus) and three different rating databases as a source for the labels (child: child ratings of child words, adult_ch: adult ratings of child words, adult: adult ratings of adult words). We will systematically test all combinations of these two factors as shown in Table 1 and evaluate their impact on the quality of the resulting computational estimates.

In Study I, we will investigate the correlations of the computational norms computed in the six conditions with human rating data. We will use rating data from existing databases from adults, but also present a new database of child ratings specifically collected for the present study.

In Study II, we will investigate the effects of the computational norms computed in the six different conditions on children’s and adults’ word processing in a lexical decision task. We will compare the effects of the various computational norms to the effects obtained using human rating data from children and adults. This allows us to determine which methodological choices approximate the effects of human ratings best.

## Study I

### Introduction

In Study I, we systematically investigate the two key variables relevant to the approach proposed by Turney and Littman (2003): The nature of the underlying VSMs and the basis for the words in the label lists (see Table 1). Regarding the nature of the underlying vector space model (child- vs. adult-specific), we expected that an adult-based vector space model will generally outperform a child-specific vector space model seen as it is trained on substantially more data (Kiela & Clark, 2014; Lapesa & Evert, 2014; Recchia & Jones, 2009; Wingfield & Connell, 2022). With regard to the different strategies for the selection of label lists the situation is less clear: Using child- or adult-specific ratings and vocabularies comes with potential (dis)advantages. Finally, it is not clear how the different sources of VSMs and label lists interact to produce appropriate norms for children.

**Fig. 1:**
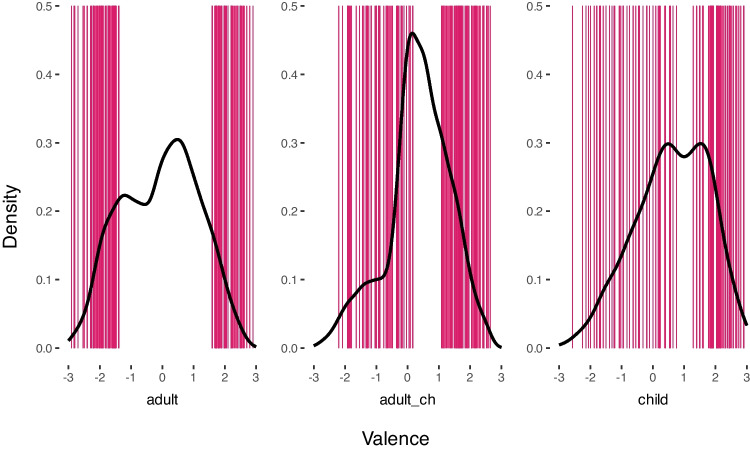
Distributions of the three rating data sets used in this study. *Left*: adult ratings of adult-specific words, *middle*: adult ratings of child-specific words, *right*: child ratings of child-specific words. Vertical lines represent the words in the label lists of positive and negative valence, generated as proposed by Lüdtke and Hugentobler (2023)

To select the different label lists, we used existing valence ratings from adults either for a relatively large subset of the adult vocabulary (i.e. ratings from an expanded BAWL database comprising 6059 words, see Methods) or for selection of child-specific words (i.e., ratings collected for the 1156 words investigated in the Developmental Lexicon Project, DeveL, Schröter & Schroeder, 2017). For children, no corresponding databases comprising a sufficient number of words are available for German at present (the largest database for children, the kidBAWL, only comprises $n = 102$ words, Sylvester et al., 2016). To compile high-quality child-specific label lists, we collected new valence ratings from children in Grade 6 for $n=535$ German words. This is currently the largest child-rating database for word valence in German, which we make available to the scientific community (see below).

### Method

#### Semantic spaces

Semantic similarities were drawn from two different VSMs. Both models were trained using gensim (Řehůřek & Sojka 2010) as a native C implementation of fastText (Bojanowski et al., 2017) with Python interfaces. fastText was chosen given its ability to account for compound words, which are particularly abundant in German, through enrichment of word-vectors with subword information (Bojanowski et al., 2017). For the child-specific space, the model was trained on childLex (Schroeder et al., 2015, 7.8 million tokens). The minimal word count was set to 2, and word embeddings were computed using a continuous bag of words (CBOW) architecture with a window size of 2 (i.e., two words 2 on either side of the target word) and a model dimensionality of 50. Subword information was retained during training. The adult-specific VSM was based on SDeWac corpus (Faaß & Eckart, 2013, 85 billion tokens). The minimal word count was set to 10, and word embeddings were computed using a skipgram architecture with a window size of 5 and a model dimensionality of 300. Subword information was retained during training. Training parameters for the adult-specific VSMs were chosen in accordance with established protocols (Baroni et al., 2014; Bojanowski et al., 2017), while selection of training parameters for the child-specific vector space was guided by recommendations presented by Günther et al., (2019).

#### Existing word rating databases

We used two existing rating databases to select the label lists for adults:

For *adult ratings of adult-specific words* we used ratings from the BAWL-R (Võ et al., 2009) ($n=2899$), which was extended with 596 words from the ANGST-database (Schmidtke et al., 2014), 1649 words from the German database from Schmidtke and Conrad (2018) and 2564 words from an extension of the BAWL-R (Conrad et al., 2014). The combined database contained 6059 words and is here referred to as “expanded BAWL”. Valence ratings were collected on a seven-point rating scale ranging from –3 (very negative) through 0 (neutral) to +3 (very positive). The mean word valence across all words was *M* = −0.07 (*SD* = 1.23, see Fig. 1).

For *adult ratings of child-specific words* we used the ratings collected in the Developmental Lexicon Project (DeveL, Schröter & Schroeder, 2017), which consist of valence, arousal, and imageability ratings for the *n* = 1152 German words in the childLex corpus (Schroeder et al., 2015). This database was used as a middle ground between adult and child databases as it contains adult ratings but features the semantic properties of a child vocabulary. Data was provided by 203 German university students (163 female, *M* age = 24.15 years, *SD* = 6.39), who rated emotional valence on a seven-point rating scale ranging from –3 (very negative) through 0 (neutral) to +3 (very positive). On average, there were 27 ratings for each word, and the reliability of the ratings was very high (ICC of.95). The mean word valence across all words was *M* = 0.35 (*SD* = 1.02, see Fig. 1).

#### Child valence ratings

As discussed above, for *child ratings of child-specific words* we collected a new rating data set. Existing databases providing valence ratings for children are very small and do not comprise a sufficient number of potential label words that cover the complete semantic dimension. In order to mitigate this, we decided to collect new rating data from children for child-specific words for which corresponding adult ratings are also available. We selected 540 words from the word list used in the DeveL project (Schröter & Schroeder, 2017). Words were selected to cover the complete valence range in the DeveL data set. Specifically, we selected 180 words with lowest and highest valence ratings, respectively, together with 180 randomly drawn words with intermediate valence (selection criteria based on existing adult ratings as described above, Schröter & Schroeder, 2017).

We decided to collect the child rating data in Grade 6 as children’s reading proficiency is high enough to perform the rating task efficiently, but their semantic representations are still reasonably child-like. For example, Sabater et al. (2020) reported a correlation of $r =.91$ between valence norms based on ratings of 9- and 11-year-old children. We aimed at collecting approximately 15 ratings for each word. This decision was based on an analysis for the rating data in the DeveL project collected from adults (see above). In that study, 6–7 ratings were sufficient to yield an ICC of .85, which is generally considered high. Assuming that data from children are twice as noisy, we estimated that approximately 15 data points are needed to obtain a similar ICC.

Overall, 84 children in Grade 6 (mean age = 12.5 years, *SD* =.6; 27 female, 11 with unrecorded gender assignment) from a local comprehensive school participated in the rating study. Data collection was approved by the Ethics board of Georg-August-University Göttingen, #316. A five-point rating scale with a smiley emoji and the anchors sad/red, slightly sad/ orange, neutral/yellow, tentatively happy/yellow-green, happy/green, as well as a question mark/grey for unknown words were presented next to each word. Children were randomly assigned a rating questionnaire that contained a total of 90 words. They were asked to indicate the positivity of each word by circling the smiley that described the word emotion best. Words that were marked as unknown by more than 50% of all raters were excluded from the data set. Raters who gave the same answer in more than 60% of all cases or knew less than 50% of the words were excluded from the data set. Data from non-native speakers were not excluded. After applying these exclusion criteria, a total of $n= 535$ words were rated by $n = 71$ (mean age = 12.5, *SD* =.6; 27 female, seven without gender assignment) children with an average number of 15 ratings per word (range [8,30]). The reliability of the ratings is high as intended (ICC of.86), but lower than for the corresponding adult ratings. Ratings were thus corrected for unreliability using the Spearman–Brown formula in all analyses. The child ratings were rescaled to the range [-3;3] in order to be comparable to the range of adult ratings. The mean word valence across all words was *M* = 0.60 (*SD* = 1.19, see Fig. 1). All rating data are provided on OSF.

#### Extraction of label lists

Label lists for positive and negative valence were generated as proposed by Lüdtke and Hugentobler (2023): words were ranked by human valence rating in descending order, then by logarithmized word frequency (based on the SDeWac, Faaß & Eckart, 2013). Next, the average rank values for each word were calculated, and the top and bottom 60 words were selected for the label list. To select labels based on *child ratings for child-specific words* (child), we used the valence ratings from our new collected child rating database. To select labels based on *adult ratings of child-specific words* (adult_ch), we used the ratings from the DeveL project (Schröter & Schroeder, 2017). Finally, to select labels based on *adults ratings of adult-specific words* (adult), we used the ratings provided by Lüdtke and Hugentobler (2023), which are based on the extended BAWL. All label lists can be found in the OSF repository for this article.

#### Calculation

We calculated norms based on the algorithm proposed by Turney and Littman (2003), which uses cosine similarities taken from a words’ corresponding vector representations from a VSM, as described in the equation section “Calculation”. In brief: The estimated valence of a word (*w*) is computed by subtracting the average cosine similarity between *w* and the negative label words ($l_n$) from the average cosine similarity between *w* and the positive label words ($l_p$; see equation section “Calculation”).

$$\begin{aligned} \text {rating}(w) = \frac{\sum _{p=1}^{i} \cos (w, l_p)}{i} - \frac{\sum _{n=1}^{j} \cos (w, l_n)}{j} \end{aligned}$$

The resulting values are bounded between -1 and 1 and were subsequently rescaled to a seven-point scale (bounded at -3 and 3).

Similarities were computed either using the *child-specific* (CHILD) or *adult-specific* vector space model (ADULT) which were trained as described above.

### Results

Overall, there were $n = 535$ words included in both our child database and the DeveL data. All analyses focus on this intersection. Ratings were corrected for unreliability using the reliabilities determined above using the Spearman–Brown formula.

First, with regard to the human rating data, the correlation between the child and the adult ratings (corrected for unreliability) was $r =.94$ and in the range of correlations between child and adult ratings that have previously been reported (e.g., $r =.90$ in Bahn et al., 2018).

Second, the intercorrelations between the computed valence norms in the six conditions are provided in Table 2. As can be seen, all correlations were positive, but differed substantially in size, varying between $r =.28$ and $r =.75$. Generally, correlations between norms derived from the same VSM were higher than the corresponding norms derived from a different VSM. The same holds true for correlations of norms derived from the same label lists across VSMs. Correlations between ratings derived from child labels were lower than correlations with ratings from adult labels. As expected, the ratings derived from the adult_ch ratings had an intermediate position and showed high correlations with both the ratings from the child and adult label list. Correlations of the combination CHILD-child were generally low.

**Table 2:** Correlations between computed norms in the six conditions

Condition	1	2	3	4	5	6
1. CHILD - child	-					
2. CHILD - adult_ch	.51	-				
3. CHILD - adult	.28	.68	-			
4. ADULT - child	.75	.57	.41	-		
5. ADULT - adult_ch	.35	.70	.44	.74	-	
6. ADULT - adult	.27	.51	.58	.63	.71	-

Note: Norms were calculated based on a child- and adult-specific VSM (CHILD vs. ADULT) in combination with label lists derived from child ratings (child), adult ratings of child vocabulary (adult_ch), or adult ratings of an adult vocabulary (adult)

In the next step, we investigated the relationship between children’s and adults’ ratings and the norms computed using either a child-specific (CHILD) or an adult-specific (ADULT) VSM, with one of three label lists (child, adult_ch, adult). Correlations with children’s ratings (corrected for unreliability) are reported in Table 3, while the correlations for adult’s ratings (also corrected for unreliability) are reported in Table 4.

**Table 3:** Correlations between computed norms and human ratings from children

	Vector Space	
Labels	CHILD	ADULT
child	.41	.68
adult_ch	.52	.59
adult	.53	.71

Note: Norms were calculated based on a child- and adult-specific VSM in combination with label lists derived from child ratings, adult ratings of child vocabulary, or adult ratings of an adult vocabulary

#### Ratings from children

For children’s ratings (Table 3), correlations with norms computed using a child-specific VSM (CHILD) were generally lower ($r =.41 -.53$) than norms computed using an adult-specific VSM ($r =.59-.71$), i.e., norms based on a larger, but unspecific VSM yielded higher correlations. With regard to the impact of the label list, norms based on adult rating norms were higher than norms based on child ratings. That is, independent of the VSM used, norms based on child ratings never showed an advantage.

**Table 4:** Correlations between computed norms and human ratings from adults

	Vector Space	
Labels	CHILD	ADULT
child	.36	.63
adult_ch	.54	.63
adult	.58	.74

Note: Norms were calculated based on a child- and adult-specific VSM in combination with label lists derived from child ratings, adult ratings of child vocabulary, or adult ratings of an adult vocabulary

**Fig. 2:**
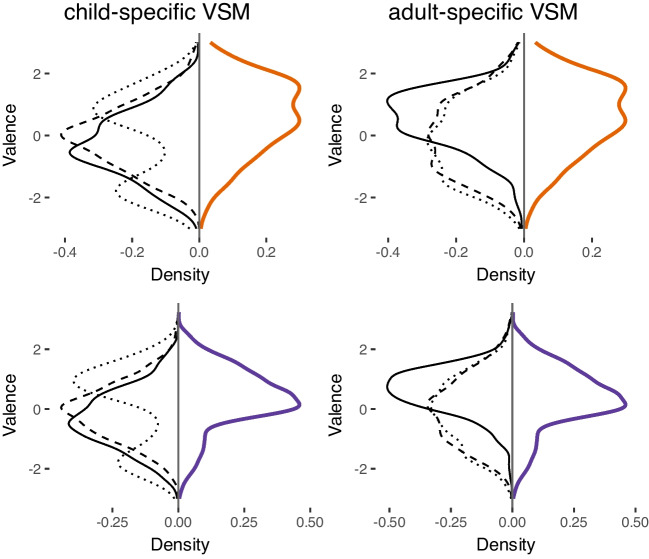
Comparison of the distributions of computed norms (*left column*: norms computed on a child-specific VSM, *right column*: norms computed on an adult-specific VSM) with human rating data (*orange*, top row: child ratings of a child vocabulary; *violet*, bottom row: adult ratings of a child vocabulary). Computed norms were based on different label lists drawn from different rating databases: *black solid lines* = norms computed based on label lists drawn from adult ratings of an adult vocabulary, *black dashed lines* = norms computed based on label lists drawn from adult ratings of a child vocabulary, and *black dotted lines* = norms computed based on child ratings of a child vocabulary

#### Ratings from adults

For adults’ ratings (Table 4), correlations with norms computed using a child-specific VSM (CHILD) were again generally lower ($r =.36 -.58$) than norms computed using an adult-specific VSM (ADULT; $r =.63-.74$). With regard to the impact of the label list, correlations of norms based on adult ratings were higher than norms generated by labels based on child ratings, especially if they were taken from the BAWL database, which covers a wider range of the valence spectrum.

Overall, it seems that label lists based on adult ratings and using adult-specific words seem to be the best choice to approximate human valence ratings, and this also holds true if the ratings are provided by children.

#### Comparison of distributions

To better understand the pattern found in the correlation analysis, we compared the distributions of human ratings to the corresponding distributions of computed norms. Here, we directed our analysis towards the question of whether the rated and computed norms have similar distributions, and additionally whether both norms span the same valence range.

With this in mind, we compared the distributions of norms computed based on a child-specific VSM (cf. Fig. 2, left column) and an adult-specific VSM (cf. Fig. 2, right column) between each other and in relation to the human rating databases (see also Fig. 1).

In brought terms, we found that the means of human ratings (violet) of child-specific words (rated by both adult-, cf. Fig. 2, top row, and child-, cf. Fig. 2, bottom row, raters) are shifted towards a more positive average valence.

A visual inspection of norms derived from both types of underlying VSMs reveals that norms derived from a child-specific VSM (cf. Fig. 2, left column) do not align with human ratings, while norms derived from an adult-specific VSM (cf. Fig. 2, right column) in general align better.

Specifically, we find that within the group of norms computed based on a *child-specific VSM*, distributions obtained using child-specific label lists (black dotted line) exhibited a bimodal pattern (refer to Fig. 2, left column, orange), while norms computed based on labels drawn from adult ratings of adult- (black solid line) and child-specific (black dashed line) vocabularies show an approximate normal distribution.

In contrast, regarding norms derived from an *adult-specific VSM* (cf. Fig. 2, right column), we not only observe a better general overlap with human ratings but also find that all norms show an approximate normal distribution. As in the correlation analysis above, norms obtained for label lists drawn from adult ratings of adult vocabularies (solid line) mirror all human ratings best.

#### Additional comparisons

In the next step, we wanted to ensure that the observed pattern of results is not driven by other properties of the underlying databases and generalizes to other approaches to compute valence norms.

First, the various databases do not only differ in the words they comprise (with words for children being positively biased), but also quantitatively in terms of the number of words that are available. In order to investigate whether the nature of the words or the size of the database is driving the observed findings, we artificially shortened the adult database by randomly drawing 1152 words (i.e., the size of the DeveL database), thereby generating a database that equals the adult_ch database in size, but still contains words from the entire adult word space (“adult small”). Next, we took the full adult database and artificially introduced a positivity bias by excluding all words with a rating < -1 (“adult bias”), and finally, we combined both manipulations by selecting 1152 words with ratings above -1 (“adult small + bias”). We computed norms based on these three manipulations using the CHILD and ADULT VSM and compared them to the results found with the full, unrestricted adult database (“adult full”, as reported above). The corresponding correlations are provided in Table 5.

**Table 5:** Correlations between human ratings from adults and computed norms upon size and bias manipulation

	Vector Space	
Labels	CHILD	ADULT
adult full	.53	.71
adult small	.55	.74
adult bias	.28	.44
adult small + bias	.28	.44

Correlations with norms computed using a child-specific VSM (CHILD) were again generally lower (*r* =.28 to .55) than norms computed using an adult-specific VSM (ADULT; *r* =.44 to .74). Correlations of norms based on the adult small manipulation with ratings from adults did not deviate substantially from the original data (adult full), while correlations significantly dropped upon introduction of a positivity bias for both VSMs.

In the final step, we wanted to check whether the reported pattern of results is specific to the Turney & Littmann algorithm or generalizes to other commonly used approaches to compute valence norms. To this end, we repeated all calculations using a k-nearest neighborhood approach proposed by Bestgen and Vincze (2012). Tables 6 and 7 provide the results for children and adults, respectively, which mirror Tables 3 and 4 above.

**Table 6:** Correlations between human ratings from children and computed norms following Bestgen’s approach

	Vector Space	
Labels	CHILD	ADULT
child	.41	.65
adult_ch	.42	.62
adult	.51	.72

Overall, the results were highly similar for the two approaches. Correlations using an adult VSM and label lists derived from adult ratings were always higher for both children and adults. Generally, the performance level of the two algorithms was similarly high, with some minor differences for individual VSM/label list combinations.

Finally, we also conducted additional analyses in order to ensure that the reported pattern of results is not driven by other differences between the child and adult data. This included the use of different rating scales (5- vs. 7-point rating scales) as well as differences in the cut-off values used to compute the child and adult VSMs. Neither of these factors had a substantial effect on the observed pattern of results.

**Table 7:** Correlations between human ratings from adults and computed norms following Bestgen’s KNN approach

	Vector Space	
Labels	CHILD	ADULT
child	.42	.62
adult_ch	.45	.66
adult	.52	.77

We also tested whether results changed if only 20 labels were used in the label list. Results were generally worse for smaller label lists, and the differences between the child and adult label lists were less pronounced. However, the combination of using an adult VSM and labels based on adult ratings still showed the best performance. The full results of all analyses can be found in supplemental materials S1 and S2 for the manuscript on OSF.

### Discussion

The central question of Study I was which combination of VSM and label-list best predicts valence ratings from children and adults.

Regarding the source of VSM, in line with our initial prediction, we find that the larger adult-specific VSM outperforms a smaller child-specific VSM. Our results align with results from other studies, which show that models trained on more data typically outperform models that have been trained on smaller training corpora (Kiela & Clark, 2014; Lapesa & Evert, 2014; Recchia & Jones, 2009; Wingfield & Connell, 2022).

Regarding the source of label lists, our results show that, overall, label lists derived from adult ratings of adult-specific words outperform label lists drawn from ratings from children. These differences cannot be attributed to differences in the resolution of the rating scales or the reliabilities of the ratings, which were taken into account in the analyses. Thus, based on the present findings, there is no need to collect ratings from children as ratings from adults for the same words perform on par or better. Moreover, our results show that using ratings from adults from a larger set of adult-specific words increases performance.

The reason for this pattern is revealed in the analysis of the distributions of human rating data (cf. Fig. 1). Distributions of child and adult_ch ratings are shifted towards the upper (positive) end of the scale. Labels derived from these databases therefore exhibit a positivity bias (mean valence on the positive label lists *M* = 2.1, *SD* = 0.4 and *M* = 1.8, *SD* = 0.44 for child and adult_ch lists, respectively and mean valence on the negative label lists *M* = −0.79, *SD* = 0.86 and *M* = −0.87, *SD* = 0.67 for child and adult_ch lists, respectively). This leads to an over-estimation of the negative term in equation Sect. “Calculation” and a left-shift in distributions, particularly when a smaller, less diverse VSM is used. Conversely, adult ratings of an adult vocabulary span the entire valence range (mean valence of *M* = 2.1, *SD* = 0.3 and *M* = −1.9, *SD* = 0.37 for positive and negative adult label lists). The inherent bias in the labels drawn from this database is thereby diminished, and the corresponding norms accurately reflect human ratings. This finding is supported by additional analyses using a smaller subset of the adult rating database with an artificially introduced positivity bias. Results show that the fact that labels derived from adult databases are less positively biased is more important to explain the observed pattern of results than differences in their size. Moreover, these additional analyses show that the present findings generalize to other approaches to compute valence norms and smaller label lists.

From a practical view, our findings are good news as they imply – rather counter-intuitively – that there is no need to use a child-specific VSM or to collect dedicated ratings from children, which are both typically more effortful to obtain. Instead, an off-the-shelf VSM based on adult data and norms from existing databases can be used to approximate valence norms for children.

However, even the best combination based on an adult-specific VSM and adult-specific labels, only yields a correlation of $r =.71$ with children’s ratings and a correlation of $r =.74$ with adults’ ratings. While there are certainly some commonalities between human ratings and computational norms, there are also important differences. Therefore, an important follow-up question is whether computational norms can reasonably substitute human rating data in their ability to predict word processing data from children and adults. We will address this question in Study II.

## Study II

### Introduction

One paradigm, which is often used to study the effect of word valence on lexical processing, is the lexical decision task, where participants have to discriminate between words and pseudowords. In adults, research has consistently shown that emotional valence influences performance in this task both in terms of accuracy and reaction times (Crossfield & Damian, 2021), indicating that valence information has an impact on word recognition processes. Most studies report an inverse U-shaped relationship between valence and reaction time (Kissler & Koessler, 2011; Kousta et al., 2009), while some studies report linear effects (Kuperman et al., 2014). In a recent meta-analysis for the lexical decision task, Ferré et al. (2025) reported substantial evidence for a positivity effect, i.e., a processing advantage of positive compared to neutral words. The corresponding negativity effect, i.e., a processing advantage of negative words compared to neutral words, was substantially smaller and not significant. For children, the situation is less clear (Conte et al., 2019): Although they seem to process positive words faster than neutral words (Lund et al., 2019), it is not clear whether they also show negativity effects.

In Study II we investigated the impact of children’s and adults’ valence ratings on reaction times collected from adults and children in a lexical decision task. In particular, we were interested in which of the valence norms computed in Study I (i.e., based on either a child or adult VSM and the three different label lists) approximates the shape of the valence effect best. The logic of the analyses is as follows: Children’s and adults’ data are analyzed separately. Within each age group, we first fit a regression model with either children’s or adults’ reaction times in the lexical decision task as a criterion and the corresponding valence ratings as a predictor variable while controlling for word length, word frequency, and orthographic neighborhood size. In the next step, we substituted the human valence ratings with the valence estimates generated by one of the six approaches investigated in Study I.

In line with the findings from Study I, we expect an advantage of norms based on an adult-specific VSM. We further expect an advantage of labels drawn from adult ratings of an adult vocabulary, again in line with Study I.

### Method

#### Behavioral data

We reanalyzed data from the Developmental Lexicon Project (Schröter & Schroeder, 2017). We selected the aggregated reaction times for the lexical decision task from children in Grade 6 (mean age *M* = 9.2, *SD* = 0.7) and from young adults (mean age *M* = 24.9, *SD* = 3.3). We used all $n = 535$ words for which both ratings from children and adults were available.

**Table 8:** Effects of word valence and lexical control variables on lexical decision times for children and adults

	Children			Adults		
	*b*	*SD*	*p*	*b*	*SD*	*p*
Intercept	6.958	0.006	<.001***	6.353	0.003	<.001***
Step 1						
Length	0.049	0.004	<.001***	0.020	0.003	<.001***
Frequency	−0.047	0.004	<.001***	−0.023	0.002	<.001***
OLD20	−0.012	0.005	.012*	0.001	0.003	.730
Step 2						
Valence (lin.)	−0.022	0.004	<.001***	−0.010	0.002	<.001***
Valence (sq.)	−0.009	0.003	<.01**	−0.006	0.002	<.01**

#### Stimuli and rating data

We used the same norm and rating data as detailed in Study I (see section “Existing word rating databases”). In addition, we controlled for word length (number of letters), word frequency (lemma frequency per million words) in the childLex corpus for children (Schroeder et al., 2015) and in the DWDS corpus for adults (Geyken et al., 2017)), and OLD20 (the mean Levenshtein distance to the 20 nearest orthographic neighbors computed using the same corpora).

**Fig. 3:**
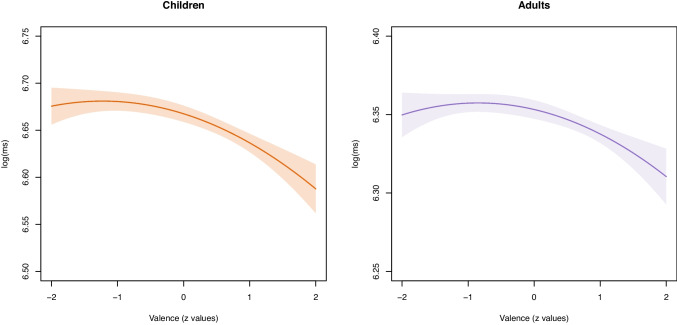
Effect of word valence on lexical decision times for **a**) children and **b**) adults

**Table 9:**
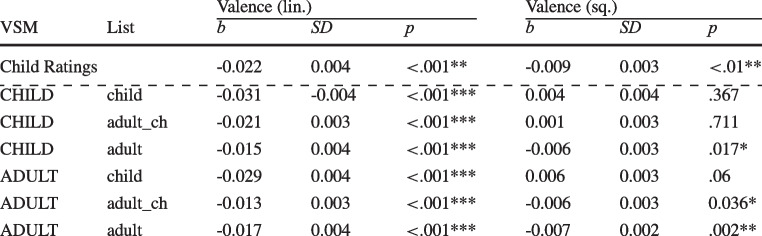
Valence effects for computational estimates in the various conditions for children

Note: Estimates were calculated based on a child- vs. adult-specific VSM in combination with label lists derived from child ratings, adult ratings of child-specific words, or adults of adult-specific words

#### Analysis

In a first analysis, we used children’s and adults’ ratings to predict lexical decision times of children and adults. We did not model response accuracy because overall response accuracy was high (*M* = 0.94, *SD* = 0.05 and *M* = 0.96, *SD* = 0.04 for Grade 6 children and adults, respectively). Response times were log-transformed prior to estimation. In order to ease interpretation, estimates were back-transformed to milliseconds. Separate models were fitted for children and for adults. In each model, scaled, age-specific variables were used (ratings from children vs. adults, lexical variables derived from child- vs. adult-specific corpora). Word length, frequency, and neighborhood size were entered as control variables in Step 1. In Step 2, both a linear and quadratic term for the valence ratings were entered.

In a second analysis, we fitted the same models for each age group, again with the control variables in a primary analysis, but now using the valence estimates from the six conditions evaluated in Study I in the second analysis. For each age group, we compared the estimates for the valence effects (second analysis) with the corresponding estimates from the human rating models (first analysis). The central question is whether the estimates in the various conditions generate valence effects that are qualitatively similar to the human rating effects.

### Results

#### Valence effects based on human ratings

Model estimates for the two blocked regression models investigating the effects of children’s and adults’ valence ratings on lexical decision latencies are provided in Table 8.

For children, the control variables entered in Step 1 were able to explain 43.4 % of the response time variance. The effects of word length and word frequency were strong and significant, while the effect of OLD20 was smaller. Entering valence in Step 2 generated a increment of 4% of the variance, which was significant, *F*(2, 531) = 20.04, $p<$.001. Both the linear and the quadratic effect of valence were significant. The effect is visualized in Fig. 3 left subplot). The effect was clearly non-linear as negative and neutral words were processed similarly slowly, while positive words were processed faster: Response times for words with valence scores 2 *SDs* below the mean, $M = 793$ ms, did not significantly differ from words with average valence scores, $M = 786$ ms; *b* = 0.008, *SE* = 0.011, *t* = 0.718, *p* =.473. By contrast, words with valence scores 2 *SDs* above the mean, $M = 726$, were processed substantially faster than neutral words, *b* = −0.79, *SE* = 0.014, *t* = −5.343, $p<$ =.001. Overall, results indicate that children showed a positivity effect, but no negativity effect.

For adults, the control variables entered in Step 1 explained 36.6 % of the response time variance. The effects of word length and word frequency were both strong and significant, while the OLD20 effect was not. Entering valence in Step 2 generated an increment of 2.8 % of the variance, which was significant, *F*(2, 529) = 12.37, $$p<$$.001. Both the linear and quadratic effects of valence were significant. The effect is visualized in Fig. 3 right subplot). Response times for words with valence scores 2 *SDs* below the mean, $$M = 572$$ ms, did not significantly differ from words with average valence scores, $$M = 574$$ ms; *b* = −0.003, *SE* = 0.009, *t* = −0.398, *p* =.691. By contrast, words with valence scores 2 *SDs* above the mean, $$M = 550 $$ ms, were processed substantially faster than neutral words, *b* = −0.043, *SE* = 0.011, *t* = −4.018, $$p<$$ =.001. Overall, adults showed similar, although weaker, valence effects than children.

#### Valence effects based on computational estimates

After having established the functional form of the valence effect for children and adults using human ratings, we turn to the main question of Study II, that is, whether computational estimates are able to capture the form of the valence effect and whether there are differences between the different ways how to compute them. The effects of the control variables entered in Step 1 were identical to the human rating models and not reported again.

For children, the linear and quadratic effects of computation valence are provided in Table 9 together with the effects generated by the human ratings in the first row to ease the comparison.

The linear effect was significant for all computational estimates. Values were generally similar to the reference effect based on human ratings, except for the estimates based on the child list, which are generally too large. The quadratic effect was only significant if an adult label list was used. For the adult-specific VSM, the effect was also significant for the adult_ch label list.

**Fig. 4:**
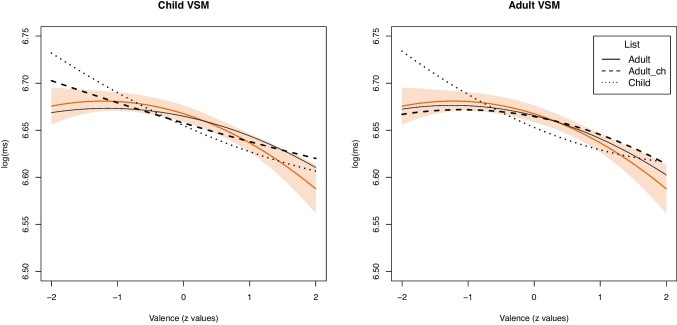
Comparisons of effects of word norms computed using **a**) a child-specific and **b**) an adult-specific VSM on lexical decision times for children

The effects of the estimates are depicted in Fig. 4. The orange line is the effect generated by the human ratings with 95% confidence intervals. The effects of the estimates based on the child-specific VSM are provided in the left subplot and the estimates based on an adult-specific VSM in the right subplot.

**Table 10:**
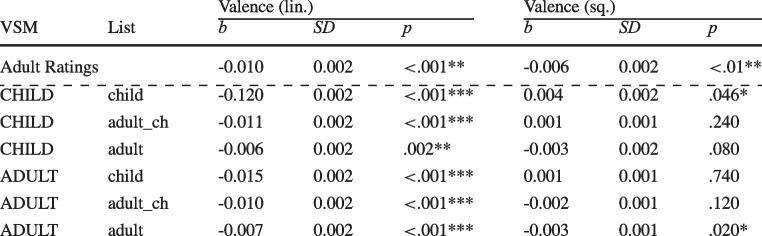
Valence effects for computational estimates in the various conditions for adults

Note: Estimates were calculated based on a child- vs. adult-specific VSM in combination with label lists derived from child ratings, adult ratings of child-specific words, or adults of adult-specific words

For the child-specific VSM, results show that estimates based on child ratings (dotted line) or adult ratings of child words (dashed line) are linear, which leads to a negativity effect that is not observed using human ratings. The effect generated by estimates based on adult ratings and adult vocabulary (solid line) is generally more reasonable and approximates the valence effect found for human ratings.

For the adult-specific VSM, estimates generated by label lists based on child ratings (dotted line), again, suggest a spurious negativity effect whereby more negative valence is associated with slower, not faster, responses, while estimates based on adult ratings (dashed line) do not. Only estimates based on adult ratings (solid line) capture the positivity effect reasonably well.

For adults, the linear and quadratic effects of computed valence norms are provided in Table 10 together with the effects generated by the human ratings to ease the comparison.

The linear effect was significant for all computational estimates. Values were generally similar to the reference effect based on human ratings. The quadratic effect was never significant when using norms based on a child-specific VSM, or even went in the opposite direction. For the adult-specific VSM, the quadratic effect was only significant and similar in size if estimates computed using adult ratings of an adult-specific vocabulary are used (adult).

**Fig. 5:**
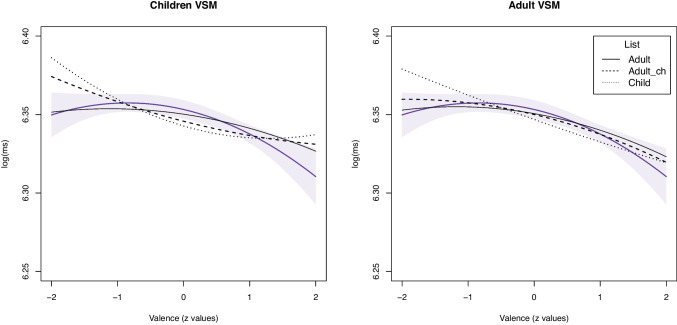
Comparisons of effects of word norms computed using **a** a child-specific and **b** an adult-specific VSM on lexical decision times for adults

The effects of the estimates are depicted in Fig. 5. The violet line is the effect generated by the human ratings with 95% confidence intervals. The effects of the estimates based on a child-specific VSM are provided in the left subplot, and the estimates based on an adult-specific VSM in the right subplot.

For both the child- and adult-specific VSM, results show that estimates computed using labels list based on child words (dashed or dotted) are not able to capture the nature of the valence effect and overestimate the negativity effect. By contrast, estimates computed using label lists based on an adult-specific vocabulary (solid line) capture the valence effect best. The effect does not significantly differ from the empirical effect, but is somewhat weaker.

We have additionally tested our word norms as a predictor for lexical decision times for younger children using the reaction times for children in Grade 2 and Grade 4 supplied in the DeveL database. The pattern reported here for children in Grade 6 emerges even more pronouncedly when data from younger children are used (detailed results are provided in the supplemental materials S3 in the OSF repository for this article). The reported pattern of findings is thus not specific to one particular age group.

### Discussion

The central aim of Study II was to evaluate the combination of VSM and label-list in their ability to approximate the effect of human valence ratings in a lexical decision task. Several points are worth highlighting:

First, our findings corroborate that there is a quadratic relationship between human ratings and reaction times in a lexical decision task. In particular, we found that negative words are processed similarly as neutral words, while positive words are processed faster. This effect is in line with the literature (Kousta et al., 2009; Vinson et al., 2014).

Second, norms computed based on adult-specific VSMs outperformed norms based on child-specific VSMs in predicting reaction times of children. In addition, computational norms based on label lists derived from adult ratings and covering the entire adult vocabulary were generally superior to norms derived from label list based on a restricted range of words, independent of whether they have been rated by children or by adults. These findings are completely consistent with the results of Study I.

Finally and most importantly, this pattern was observed for both children and adults. That is, using norms based on an adult VSM and using a label list based on an adult vocabulary were not only better to approximate valence effects of adults, but also in children. Specifically, norms computed based on child-specific VSMs or using a child-specific label list, incorrectly predict that children and adults show a linear valence effect, that is, negative words should be processed more slowly than neutral words. Although the analyses were based on data from children in Grade 6, the same pattern was also observed in Grades 2 and 4. This indicates that the reported findings generalize to children’s word processing across a wide range of age groups.

In sum, our results indicate computational estimates of valence norms for children should be based on existing resources for adults, that is, existing VSMs based on large corpora and valence ratings from adults. By contrast, using child-specific VSMs or collecting rating data from children is not only not beneficial, but – especially when using smaller, but less precise VSMs for children – actually detrimental. We will elaborate on the underlying reasons for this apparent discrepancy next in the General discussion.

## General discussion

In the present paper, we investigated whether computational approaches can be used to approximate valence ratings from children and, if so, which methodological choices are optimal for the approximation process. In particular, we investigated a) whether a child- or adult-specific VSM should be used and b) whether the label list used in these algorithms should be based on ratings from adults or children. We conducted two studies to evaluate which of the various combinations best allows to approximate of children’s and adults’ ratings: In Study 1 we directly compared the estimates from the various conditions with ratings from children and adults and in Study 2, we evaluated which combination most appropriately approximates the functional form of children’s and adults’ valence effect in a lexical decision task. In both studies, we consistently found that computational norms based on a VSM for adults outperformed norms computed using a VSM for children. In addition, we found that label lists based on adults’ ratings and using the entire adult vocabulary performed best. Thus, somewhat paradoxically, the best way to generate computational norms for children is to entirely ignore that the norms are supposed to be used with children. In the following, we will elaborate on the theoretical and practical implications of these findings.

### Global findings

While human ratings and computational norms share some similarities, they also have important differences, and computational norms are intrinsically limited in their predictive power and accuracy (for an in-depth discussion of limitations, see Snefjella and Blank, 2020). Consistent with this, even the best estimates correlated only with $r =.66$ with children’s ratings and $r =.77$ with adult’s ratings.

While adult ratings are in line with correlations found for other computational German valence norms in literature ($r =.8$, e.g., Köper & Schulte im Walde, 2016; Palogiannidi et al., 2015), correlations with children’s ratings are somewhat lower although in a similar range as correlations found elsewhere (Bestgen & Vincze, 2012; Westbury et al., 2006, found correlations of $r=.71$ and $.37>r<.58$, respectively, with English valence ratings).

More importantly, our findings indicate that norms derived from an adult-specific VSM and label lists based on adult ratings of an adult vocabulary provided the best norms in terms of both correlation and predictive power.

To understand this pattern better, we need to revisit the algorithm proposed by Turney and Littman (2003): equation Section “Calculation” employed for bootstrapping valence norms is comprised of two separate terms. The first term represents the sum of similarities to positive concepts, and the second term the sum of similarities to negative concepts. Any skewness, be it in the label lists or the corpora, therefore propagates to the estimates in either term, and thus yields skewed corresponding norms. In the following, we will explore the individual impacts of skewness in the VSMs and label lists individually.

### Impact of the VSM

We found that norms based on an adult-specific VSM outperformed all norms based on child-specific VSMs in terms of correlations with human ratings and prediction of reaction times in a lexical decision task. In line with current findings on big-data approaches in the literature (Kiela & Clark, 2014; Lapesa & Evert, 2014; Recchia & Jones, 2009; Wingfield & Connell, 2022), data-size appears to be the most important factor, outperforming data-specificity and yielding better quality norms in terms of their respective correlations with human ratings

In particular, child corpora suffer from two fundamental limitations: on the one hand, they are inherently smaller in size, on the other hand, child corpora are characterized by a focus on more positive topics. The first limitation translates to a more inaccurate representation of the semantic space which is driven, in part, by the size in terms of *token*, translating to fewer instances to learn from and consequently leading to an inferior representation of semantic organization, much like a VSM computed with fewer epochs will be less accurate (Mikolov et al., 2013, 2017). Additionally, the relative scarcity of *types* may lead to a misrepresentation of the actual semantic space and an unnatural clustering of concepts in space. As a consequence, emotional categories are more blurred in a child-specific VSM, thus leading to a skewness in semantic similarities which then propagates to the algorithm proposed by Turney and Littman (2003) and falsely informs the corresponding norms.

The second limitation leads to a positivity bias in the semantic space: in other words, words that are comparably neutral make up the negative end of concepts in child-specific corpora, and child corpora are usually devoid of extremely negative terms such as famine or death (Jacobs et al., 2020). As a consequence, the semantic space based on a child corpus is distorted towards a more positive pole, and similarities between seemingly negative (in a child-verse) terms and *positive* labels are informed by an artificial positivity inherent to the VSM. According to the logic of the algorithm proposed by Turney and Littman (2003), the first component (i.e., the sum of similarities to positive concepts) is overestimated. This leads to an underestimation of the total valence for negative concepts, making them appear more positive. Consequently, this exacerbates the skewness, particularly on the negative side.

Based on these theoretical considerations, we can conclude that norms based on child VSMs inadequately cover the negative valence range and therefore do not correlate well with human rating data. They further perform poorly as predictors of human reactions to verbal stimuli. In both cases, correlations with human ratings and prediction of human behavior, the effects are driven by the negative value range.

### Impact of the label lists

In addition to the effects found for the VSM, we find an advantage of adult-specific label lists. Labels derived from adult ratings of an adult vocabulary enhance the overall performance of the norms, improving both correlations with human ratings and their predictive power for reaction times in a lexical decision task. In contrast, labels derived from either adult or child ratings of a child’s vocabulary perform less well.

This effect, again, is driven by the under-representation of negative information. The items used in rating studies with children do usually not use very negative instances and thus have an inherent positivity bias (which can also be observed in our data, please cf. Fig. 1). Consequently, the negative labels drawn from a database representing a child vocabulary do not represent the true negative range of valence but instead are more representative of the negative-to-center section of a typical valence range. Within the logic of the algorithm proposed by Turney and Littman (2003), we would ideally expect negative target words to be very similar (semantic similarity $\rightarrow$ max) to negative labels and likewise very dissimilar (semantic similarity $\rightarrow$ min) to positive labels. If, however, the negative labels do not represent the true negative pole, but instead are more representative of the center of the valence range, then the loading of the separate terms within the algorithm will switch: semantic similarities of a negative target word with positive labels will increase ($\rightarrow$ max), while semantic similarities with negative labels will decrease ($\rightarrow$ min). The positive term within the algorithm is thus grossly overestimated, while the negative term is underestimated, skewing the computed valence range towards the positive scale.

In a similar manner as observed for the different sources of VSMs, norms generated using labels drawn from a child’s vocabulary inadequately cover the negative valence range. As a consequence, computed norms based on child-specific vocabularies globally fall short in their correlations to human ratings and do not accurately predict the quadratic relationship between word valence and reaction times in a lexical decision task. This effect can in both cases be attributed to an under-representation of the negative value range, where, for reasons given above, norms computed using labels drawn from child-specific vocabularies fail to approximate the expected behavior.

## Conclusions and follow-up questions

In conclusion, we demonstrated that the specificity of either VSM or label lists does not increase model performance, but instead has a detrimental effect on the computation of word norms for children. Based on our data, we recommend using off-the-shelf VSMs readily available online (Mikolov et al., 2017). Moreover, the collection of child-specific ratings to generate label lists does not provide an advantage over adult ratings and laborious collection of such rating data from children can be omitted in favor of adult ratings, which are more easily obtained and already exist for many languages. In combination, both data sources allow researchers to compute word norms for children with high criterion-related validity (high correlations with human rating data, cf. Study I) and high construct validity (very good approximation of the relationship found for human valence ratings and reaction times in a lexical decision task, cf. Study II).

Our results indicate that the present findings are not specific to the Turney and Littman approach, but generalize to label lists of different sizes and other computational approaches such as Bestgen’s k-nearest neighborhood algorithm. Moreover, additional analyses indicate that the reported pattern of results cannot be explained by the differences between children and adults in the type rating scales, the reliability of ratings, or the cut-off scores that were used for the computation of the VSMs. Most importantly, the results from Study II show that the present results are likely to generalize across a wider range of age groups, as the same pattern of results was found for Grades 2, 4, and 6. However, the ratings used in the present study were just collected from children in Grade 6. Although valence ratings in different age groups are usually highly correlated (Monnier & Syssau, 2017; Sabater et al., 2020), future studies should explicitly test whether different results are obtained if rating data are collected from younger children.

While there are existing child rating datasets for word valence, researchers interested in exploring the effects of other semantic dimensions, such as arousal or imageability, currently face challenges. Adult norm research has demonstrated that the algorithm proposed by Turney and Littman (2003) can effectively be adapted to various semantic variables (Köper & Schulte im Walde, 2016). Therefore, a natural extension of our findings involves applying our approach to other semantic variables for the computation of norms that are suitable for predicting child behavior.

Similarly, this methodology could be extended to other languages facing comparable shortages of child-appropriate norms. Here, we have shown that using a rating database of adult ratings that appropriately covers the entire range of a semantic category is suitable for drawing labels for the computation of German valence norms. Consequently, even from a relatively modest initial database of human ratings, labels can be derived to facilitate the calculation of child-appropriate norms. While direct translation of labels and ratings inevitably involves some loss of nuance (Charbonnier & Wartena, 2020), the broader semantic trends remain consistent across languages. As such, researchers may opt to utilize translations of the labels employed in this study as an alternative approach.

It is worth broadening our perspective regarding the underlying semantic model. This prompts two questions:

Firstly, one might ask whether augmenting the VSM architecture with transformer-based models, alongside adjustments to the underlying corpus (Yang et al., 2023), could yield an additional enhancement in norm quality. However, while transformer-based models excel at more complex tasks like text generation (Amini et al., 2023; Wiseman et al., 2017) or machine translations (Bojar et al., 2017), the algorithm proposed by Turney and Littman (2003) relies on type-level semantic representations. Previous research has indicated that word-to-vector architectures perform at least at par with transformer-based representations for this kind of application (Cassani et al., 2023; Lenci et al., 2022).

Moreover, the primary distinction between most adult-specific VSMs and our child VSM is that the adult VSMs encompass a broader range of sources, such as newspaper articles and web pages, in addition to literature (Faaß & Eckart, 2013). This broader representation of language input among adults helps to counteract the positivity bias inherent in literary sources. This further helps to explain the superiority of corpora such as the SDeWaC (Faaß & Eckart, 2013) in generating reliable norms, and underlines the applicability of large, diverse corpora to compute norms even for a child population. In the future, it would be worthwhile to replicate this study using a similar corpus for children, which includes not only literature but also transcripts of real conversations (e.g., using CHILDES) and other diverse language inputs to obtain a more balanced representation of valence in the corpus itself.

Additionally, we need to consider that not all children are the same, and while a 2-year age difference may only slightly affect cognitive states in adults, it can have a stronger impact in a younger population due to pronounced developmental changes taking place at that age. Therefore, from a theoretical point of view, the use of corpora specifically tailored to a reading age would be preferable. However, specificity comes at the cost of data size: a comprehensive corpus containing texts aimed at a broader age range (e.g., childLex, Schroeder et al., 2015) offers a sufficiently large dataset to reliably generate a VSM. Nonetheless, we are aware that the use of comprehensive corpora may sacrifice nuanced, age-specific details, thereby potentially diminishing the overall specificity of the norms.

Finally, building on the idea of specialist corpora, a departure from contemporary literature corpora towards collections focused on historical (Davies, 2012; Lin et al., 2012) or theme-specific domains (such as law or biochemistry) could facilitate the generation of norms customized for these particular fields (see e.g., Buechel et al., 2016, for norms informed by language in historical texts). Although definitive evidence supporting this application is presently lacking (and we encourage other researchers to explore this avenue further), our data indicates that a sufficiently large corpus, alongside a rating database spanning the entire range, remains the most reliable predictor, even when establishing norms for niche domains.

## Supplementary information

All supplementary materials for this article are available on the OSF repository: https://osf.io/vkzhb/. These include the child valence rating database, analyses of the effects of VSM cut-off values (S1), resolution effects between rating databases (S2), and detailed results on the generalizability of our findings, including additional data from children in Grades 2 and 4 (S3).

## Data Availability

The valence rating database of child ratings is made available on the OSF repository for this article.

## Footnotes

1 Rating data are not a linear combination of their constituents: Abend (evening): valence = 1.6, rot (red): valence = 1.0 Abendrot (evening glow): valence = 2.4; data taken from BAWL-R (Võ et al., 2009).

## Code availability

The code for generation of VSMs as well as for the computation of norms is made available on the OSF repository for this article.
